# The first psychotic episode with religious delusion in adolescence and young adults: premanifest stage

**DOI:** 10.1192/j.eurpsy.2022.2000

**Published:** 2022-09-01

**Authors:** V. Kaleda, U. Popovich, N. Romanenko, E. Gedevani

**Affiliations:** 1 FSBSI «Mental Health Research Centre», Department Of Youth Psychiatry, Moscow, Russian Federation; 2 FSBSI «Mental Health Research Centre», Researching Group Of Specific Forms Of Mental Disorders, Moscow, Russian Federation

**Keywords:** young age, religious delusion, manifest psychotic episode, religiosity in premorbid

## Abstract

**Introduction:**

The study of the premanifest stages of the first psychotic episode with religious delusion is relevant due to the lack of clarity in differentiating normal religiosity from pathological and, as a consequence, a relatively longer period of an untreated psychotic state, which negatively affects both the course of the disease and its outcomes. Another important factor is the high risk of antisocial, autoaggressive and suicidal behavior at different stages of the disease.

**Objectives:**

The aim of the study is to identify the conditions for the formation of religious delusion in adolescence and young adults, to analyze the correlations between religiosity at the pre-manifest stage and the subsequent manifest psychotic episode with religious delusions of different content.

**Methods:**

The 57 male patients at a young age (16-25 years) with a manifest psychotic episode (F20, F25 according to ICD-10) with religious delusion (delusion of sin (21,6 %), delusion of demonic possession (29,4 %), antagonistic and messianic delusion (39,2 %), oneiroid with religious content (9,8 %)) were studied with the clinical-psychopathological, psychometric (PAS) methods. The religiosity of patients in premorbid was assessed with the Duke University Religion Index (DUREL) questionnaire.

**Results:**

Of greatest importance in the formation of psychotic episode with religious content is hereditary burden, premorbid personality structure, high scores on the PAS scale.

**Conclusions:**

The presence or absence of religiosity in premorbid doesn’t matter to formation of psychotic episode with religious delusion.

**Disclosure:**

No significant relationships.

